# Histological reclassification of parotid gland carcinomas: importance for clinicians

**DOI:** 10.1007/s00405-016-4048-8

**Published:** 2016-04-21

**Authors:** Dominik Stodulski, Hanna Majewska, Alena Skálová, Bogusław Mikaszewski, Wojciech Biernat, Czesław Stankiewicz

**Affiliations:** 1 Department of Otolaryngology, Medical University of Gdańsk, Gdańsk, Poland; 2 Department of Pathomorphology, Medical University of Gdańsk, Gdańsk, Poland; 3 Department of Pathology, Charles University in Prague, Faculty of Medicine in Pilsen, Pilsen, Czech Republic

**Keywords:** Parotid gland carcinoma, Histopathology, Revision, Clinical course

## Abstract

Reassessment of histological specimens of salivary gland carcinomas is associated with a change of primary diagnosis in a significant number of patients. The authors evaluated the relation between reclassification/verification of histological diagnosis and the clinical course of parotid gland carcinomas. Histological and immunohistochemical examinations of 111 specimens of parotid gland carcinomas operated on during the years 1992–2010 were revised and in some cases supplemented with cytogenetic tests (FISH), to verify the diagnosis and potentially reclassify the tumours. Analysis of the clinical documentation and follow-up data of patients whose diagnosis was changed was then carried out. The prognostic factors taken into account in the evaluation of the clinical course included the T and N stage, the tumour grade and the extent of resection. The primary diagnosis was changed on review in 28 patients (25.2 %). In 16 patients, the change involved a different histological type of cancer. In six cases, what was thought to be a primary salivary gland cancer was reclassified as a secondary tumour. In four other cases, the change was made from a malignant to a benign tumour and in one case to a non-neoplastic lesion (necrotizing sialometaplasia). Additionally, in two patients with carcinoma ex pleomorphic adenoma, the malignant component was found to be of in situ type. A potentially atypical clinical course was observed in 4 out of 28 patients whose diagnosis was changed. In the case of 2 patients, the course of disease was more aggressive (dissemination, death) than predicted and less aggressive in rest of the patients. Histological reclassification/verification of parotid gland carcinomas can explain the cause of an atypical clinical course in some patients and sometimes enables doctors to implement a change in therapy.

## Introduction

Parotid gland carcinomas constitute a very heterogeneous group of cancers. Their diagnosis is made based on their morphological, cytological and biological (clinical) features [1].

Histological classification of salivary gland tumours by WHO undergoes constant changes. During the first edition dated 1972, 7 types of carcinomas were distinguished, in 1991 there were 18 of them and the currently binding edition from year 2005 lists as many as 24 types of carcinomas [2–4]. In future editions, the number of types of salivary gland carcinomas will probably increase. Introduction of new types of cancers is associated both with difficulties in qualifying them to previous categories based on their morphological features (histological) and the immunohistochemical phenotype, in specifying the criteria of diagnosis and in introducing new techniques (for example, genetic tests for confirmation of a given mutation) [5]. According to the literature, after a reassessment of histological specimens of salivary gland carcinomas, primary diagnosis may be changed in up to 1/3 of patients [6, 7]. Reclassification can involve diagnosis change not only from one type of cancer into another of the same or different grade but also from a primary to a secondary (metastasis) tumour or from a malignant to benign lesion [8]. The last two situations are especially associated with clinical implications due to a risk of overdiagnosis and overtreatment or underdiagnosis and an inadequate treatment. For this reason, the authors of this article have evaluated the relation between reclassification/verification of histological diagnosis and the clinical course of parotid gland carcinomas in a large case series.

## Materials and methods

Histological and immunohistochemical reassessment of 111 specimens of parotid gland carcinomas from resections performed at the Department of Otolaryngology of Medical University of Gdańsk during the years 1992–2010 was conducted in the Department of Pathomorphology of the Medical University of Gdańsk, Poland, to verify the diagnosis and potentially reclassify the tumours. Concurrently, the same specimens were independently examined and analysed cytogenetically at the Department of Pathology of the Charles University in Prague, Faculty of Medicine in Pilsen, Czech Republic. In case of differences in the assessment of specimens between the two centres, the examination was repeated and a consensus diagnosis was established. Reclassification was carried out according to histological classification by WHO from year 2005 and with addition of some new histological types of salivary gland neoplasms described since then [4, 5, 9]. Diagnoses were changed retrospectively based on microscopic appearance as interpreted by a pathologist experienced in salivary gland neoplasms, and taking into consideration clinical/follow-up data, additional immunohistochemical (IHC) and cytogenetic tests such as fluorescent in situ hybridization (FISH). Specifically, FISH was used to confirm the presence of ETV6-NTRK3 translocations in cases of *mammary analogue secretory carcinoma* (MASC) and CTRC1-MAML2 and CTRC3-MAML2 translocations in *mucoepidermoid carcinoma* (MEC) using previously described methodology [9, 10]. The prognostic factors taken into account in evaluation of the clinical course included the stage, grade and margins status. Clinical stage was based on TNM of 2009 [11].

## Results

### Histological analysis

The primary diagnosis was changed in 28 of the 111 patients (25.2 %). In 16 of those patients, the change involved reclassification of the salivary carcinoma from one type to another, and specifically, in 6 cases the change was to a new type of salivary gland carcinoma not recognized in the 2005 WHO classification—*mammary analogue secretory carcinoma* (MASC)—based on the presence of ETV6-NTRK3 translocation. In another four cases, diagnosis was changed to *salivary duct carcinoma* (SDC), supported by positive expression of HER-2 protein. Two additional patients originally diagnosed with *carcinoma ex pleomorphic adenoma* (CxPA) on review were found to have in situ carcinoma arising in *pleomorphic adenoma* (CxPA in situ). In six cases, primary cancer of salivary gland was reclassified as a secondary tumour (metastases from the kidney, breast or skin), while in four other cases the diagnosis of carcinoma was changed to a benign neoplasm (adenoma) and one case to a non-neoplastic lesion (*necrotizing sialometaplasia*). The 28 diagnostic changes are summarized in Table 1.

**Table 1:** Methods and basis for reclassification of 28 parotid gland carcinomas

Patient no	Primary diagnosis	Revised diagnosis	Methods and basis for reclassification	
1	MEC	MASC HG	FISH	Translocation ETV6-NTRK3
2	ACa NOS	MASC HG	FISH	Translocation ETV6-NTRK3
3	ACa NOS	MASC LG	FISH	Translocation ETV6-NTRK3
4	AcCC	MASC LG	FISH	Translocation ETV6-NTRK3
5	AcCC	MASC LG	FISH	Translocation ETV6-NTRK3
6	Papillary CAC	MASC LG	FISH	Translocation ETV6-NTRK3
7	UCa	NCa	IHC	Chromogranin+, CD56+, synaptophysin+, TTF1−, S100−, CK20−, CK7−
8	SCC G2	SDC	IHC	AR−, HER2+, CK7+, p63−, S100−
9	CxPA	SDC	IHC	AR+ (20 %), HER2+, CK7+
10	MEC HG	SDC	IHC	AR+, HER2+, CK7+, p63−, S100−, EMA+
11	MEC HG	SDC	IHC	AR−, HER2+, CK7+, p63−, S100−, EMA+
12	ACa NOS	AcCC	IHC	CK8+, CK7−, PAS+, DOG1+
13	AdCC	AcCC	IHC	DOG1+, PAS+
14	MEC	EMCa LG	IHC	P63+, CK7+, CK14+, calponin focally+
15	BCAca	EMCa LG	IHC	P63+, CK7+, CK14+, calponin+
16	AcCC	EMCa LG	IHC	P63+, CK7+, CK14+, calponin+
17	CxPA (SDC)	CXPA in situ	H&E	
18	CxPA (ACa NOS)	CXPA in situ	H&E	
19	CxPA	PA with SCM	H&E	Lack of atypia
20	MEC	PA with SCM	H&E/FISH	Lack of translocation CTRC1-MAML2/CTRC3-MAML2
21	Clear cell Ca	Myoepithelioma (clear cell variant)	IHC	S100+, SMA+, calponin+, GFAP+
22	MEC	Metaplastic WT	H&E/FISH	Lack of translocation CTRC1-MAML2/CTRC3-MAML2
23	PLGA	*Necrotizing sialometaplasia*	H&E	
24	MEC	SCC metastases (skin)	H&E/clinical data/follow-up	PAS−, mucicarmine−
25	Clear cell Ca	RCC metastases	IHC/clinical data/follow-up	CD10+, RCC+
26	AcCC	RCC metastases	IHC/clinical data/follow-up	CD10+, RCC+
27	AcCC	RCC metastases	IHC/clinical data/follow-up	CD10+, RCC+
28	AcCC	BC metastases	IHC/clinical data/follow-up	Mammaglobin +

*MASC* mammary analogue secretory carcinoma, *SDC* salivary duct carcinoma, *ACA NOS* adenocarcinoma not otherwise specified, *CxPA* carcinoma ex pleomorphic adenoma, *MEC* mucoepidermoid carcinoma, *SCC* squamous cell carcinoma, *AcCC* acinic cell carcinoma, *AdCC* adenoid cystic carcinoma, *EMCa* epithelial–myoepithelial carcinoma, *UCa* undifferentiated carcinoma, *PLGA* polymorphous low-grade adenocarcinoma, *NCa* neuroendocrine carcinoma, *CAC* cystadenocarcinoma, *RCC* renal cell carcinoma, *BC* breast carcinoma, *WT* Warthin tumour, *PA* pleomorphic adenoma, *SCM* squamous cell metaplasia, *HG* high grade, *IG* intermediate grade, *LG* low grade, *H&E* hematoxylin and eosin, *IHC* immunohistochemistry

### Clinical analysis

Table 2 presents clinical and histopathological data of patients, whose diagnoses were revised. A potentially atypical clinical course was observed in 4 out of 28 patients, whose diagnoses were changed. In 2 of those 4 patients (no. 25 and 26), the course of the neoplastic disease was more aggressive (generalized neoplastic disease and death) than would be predicted based on original histological diagnosis, low grade, stage, and completeness of surgical excision. In both cases, the parotid gland tumour turned out to be a metastatic lesion on review. In 2 other patients (no. 17 and 21), the situation was opposite—unexpected asymptomatic course lasting for many years after tumour resection with uncertain margin. In the first case, the lesion was rediagnosed as “in situ” *carcinoma ex pleomorphic adenoma* and in the second case, as a non-malignant neoplasm (a rare variant of *myoepithelioma*).

**Table 2:** Clinical course and follow-up of patients with revised diagnosis

No	Age	Sex	Primary histology	Grade	TNM	Resection	Revised histology	Status/years
1	60	M	MEC	IG	pT4aN1	R1 + RT	MASC HG	DOD/1
2	73	M	ACa NOS	HG	pT3N0	R1 + RT	MASC HG	L/NR/2,3,4
3	63	M	ACa NOS	LG	pT3N0	R1 + RT	MASC LG	NED/9
4	51	K	AcCC	LG	pT2N0	R0	MASC LG	NED/7
5	75	K	AcCC	LG	pT3N0	R1	MASC LG	NED/5
6	42	K	Papillary CAC	LG	pT2N0	R0	MASC LG	NED/9
7	46	K	UCa	IG	pT2N0	R0	NCa IG	NED/20
8	71	K	SCC	HG	pT3N2b	Rx + RT	SDC HG	DOD/3
9	57	M	CxPA	HG	pT4aN1	R1 + RT	SDC HG	DOD/1
10	67	M	MEC	HG	pT4aN2b	R1 + RT	SDC HG	DOD/2
11	47	K	MEC	HG	pT4aN2b	Rx + RT	SDC HG	NED/20
12	48	K	ACa NOS	IG	pT2N0	R0	AcCC LG	NED/19
13	62	M	AdCC	HG	pT2N0	R0	AcCC HG	LNR/3
14	75	M	MEC	IG	pT2N0	R0	EMCa LG	NED/6
15	52	K	BCAca	LG	pT2N0	R1 + RT	EMCa LG	LR/2,4,9
16	67	M	AcCC	LG	pT2N0	R0	EMCa LG	NED/15
17	42	M	CxPA (SDC)	HG	pT3N0	Rx	CxPA in situ	NED/15
18	51	M	CxPA (ACa NOS)	HG	pT2N0	R0	CxPA in situ	NED/9
19	40	K	CxPA (ACa NOS)		pT2N0	R0	PA with SCM	NED/17
20	71	K	MEC	IG	pT2N0	R0	PA with SCM	DOC/10
21	42	K	Clear cell Ca	LG	pT2N0	Rx	Myoepithelioma	NED/9
22	56	M	MEC	LG	pT2N0	R0	Metaplastic WT	NED/9
23	35	K	PLGA	LG	pT2N0	R1	Necrotizing sialometaplasia	NED/5
24	70	M	MEC		pT2N1	R0; RT	SCC metastases (skin)	NED/6
25	68	M	Clear cell Ca	LG	pT2N0	R0	RCC metastases	DOD/2
26	65	K	AcCC	LG	pT1N0	R0	RCC metastases	DOD/2
27	76	K	AcCC	LG	pT2N0	Rx	RCC metastases	AWD/4
28	75	K	AcCC	LG	pT1N0	R0	BC metastases	NED/10

*MASC* mammary analogue secretory carcinoma, *SDC* salivary duct carcinoma, *ACA NOS* adenocarcinoma not otherwise specified, *CxPA* carcinoma ex pleomorphic adenoma, *MEC* mucoepidermoid carcinoma, *SCC* squamous cell carcinoma, *AcCC* acinic cell carcinoma, *AdCC* adenoid cystic carcinoma, *EMCa* epithelial–myoepithelial carcinoma, *UCa* undifferentiated carcinoma, *PLGA* polymorphous low-grade adenocarcinoma, *NCa* neuroendocrine carcinoma, *CAC* cystadenocarcinoma, *RCC* renal cell carcinoma, *BC* breast carcinoma, *WT* Warthin tumour, *PA* pleomorphic adenoma, *SCM* squamous cell metaplasia, *HG* high grade, *IG* intermediate grade, *LG* low grade, *AWD* alive with disease, *NED* no evidence of disease, *DOD* died of disease, *DOC* died of other cause, *LR* local recurrence, *NR* nodal recurrence, *RT* radiation therapy, *R1* microscopically positive margin, *Rx* microscopically uncertain margin, *R0* microscopically negative margin

## Discussion

For many clinicians, a change of histological diagnosis represents a certain taboo. In our series, the primary histopathological diagnosis was changed in about a quarter of patients. We were able to find only a few other reports discussing this issue. Van der Wal et al. reassessed specimens of tumours of small salivary glands and of the parotid gland, which resulted in change of diagnosis in 29 and 11.7 % of patients, respectively. In that series, histological verification and reclassification was based exclusively on a repeat microscopic examination of the specimens. It is worth to point out that after histological revision, the diagnosis of 7 adenomas was changed to carcinoma (total in intraoral and parotid location): (2 *polymorphous low*-*grade adenocarcinoma,* 2 MEC, *adenoid cystic carcinoma*, *epithelial*–*myoepithelial carcinoma*, *malignant myoepithelioma*), and 6 cancers (2 MEC, 2 *adenoid cystic carcinoma*, CxPA, *adenocarcinoma not otherwise specified*) to adenomas and cyst [6, 7]. In the study presented by Godballe et al., in which 85 parotid gland carcinomas were reanalysed, diagnosis was changed after microscopic reassessment and immunohistochemical tests in 20 patients (23.5 %) [8]. In a large national study in Denmark, a revision of 886 cancers of the large and small salivary glands was done and diagnosis was changed in 121 of them (14 %). In 11 cases, the diagnosis was changed from carcinoma to adenoma, in 7 CxPA in situ was diagnosed, and in next 12 cases cancers appeared to be non-epithelial malignant tumours, and in 90 cases a subtype of cancer was changed. In one case, it was found that the cancer does not originate from the salivary glands [12]. Histological assessment of salivary gland neoplasms is difficult and requires specialist experience to avoid diagnostic traps, such as misdiagnosis of *necrotizing sialometaplasia* or squamous metaplasia within *Warthin tumour* as carcinoma [10, 12, 13]. Moreover, due to significant progress in the adjunct diagnostic procedures, diagnosis of parotid gland carcinomas may require immunohistochemical and molecular tests. Nowadays, many types of cancers of the salivary glands (*adenoid cystic carcinoma, epithelial*–*myoepithelial carcinoma*, MEC, MASC, *hyalinizing clear cell carcinoma*, CxPA, SDC, *acinic cell carcinoma*) have specific molecular biomarkers, which are used to confirm the diagnosis and also have prognostic significance [14]. Interesting results were presented by Bishop et al. After microscopic and immunohistochemical re-evaluation, and after applying molecular techniques (FISH) to *acinic cell carcinoma* (AcCC) specimens, the diagnosis was changed to an MASC in 9/11 (82 %) of tumours in intraoral location, 2/2 in submandibular gland, and only in 3 of 16 (19 %) in parotid [15]. Another problem faced by nonspecialist pathologists is lack of awareness of newly defined salivary neoplasms such as mucinous variant of *myoepithelioma* and MASC, as discovered in our study. This is illustrated in our study by the relatively high number of patients, whose diagnoses were changed from primary carcinoma of a salivary gland to a secondary lesion (metastasis to the parotid gland from kidneys, breast or skin). This is similar to the findings of Godballe et al., who reported revision of primary carcinomas to metastatic ones in 6 % of patients, with the primary location of the tumour in the breast, prostate, skin and lung [8]. Metastases to the parotid gland make 5–11 % of all malignancies of this gland with the vast majority of them originating in the skin on the head (*squamous cell carcinoma* and *malignant melanoma*) [16–18]. However, occasionally, the primary malignancy is located outside the head and neck (kidney, breast and lung), and the metastatic tumour can be its first symptoms [18, 19]. This illustrates why access to full clinical data is necessary for proper diagnosis [16–19].

Predicting the clinical course based on histology and progression of the disease is not obvious and to a great extent is subjective. In the studies by Van der Wal et al., during further follow-up of patients after histological reclassification there were no events observed to confirm the accuracy of diagnosis change [6, 7]. A change in the diagnosis from a malignant neoplasm to a benign one, a non-neoplastic lesion or an in situ cancer (CXPA), has a psychological significance for the patient; however, the practical (economic) aspect is important as well (shortening/conclusion of follow-up). Moreover, a change in diagnosis can occasionally enable new therapeutic options such as use of monoclonal antibody treatment (*Trastuzumab*, *Cetuximab*), kinases inhibitors BRAF, MTOR, MEK, androgen receptor blockers and others [14, 20].

## Conclusions

Histological assessment of salivary gland carcinomas should be carried out by an experienced pathologist with an access to a specific panel of IHC and molecular tests. It is also crucial for pathologists to have access to patients’ full clinical data, especially the information about past treatment of other primary neoplasms. Histological reclassification/verification of parotid gland carcinomas can help explain the cause of atypical clinical course in some patients, and may sometimes enable clinicians to implement proper therapy at early stages of the disease.
